# Parotid Oncocytomas Express the Glycoprotein Non-metastatic Melanoma Protein B (GPNMB): A Potential Link with Birt-Hogg-Dubé Syndrome

**DOI:** 10.1007/s12105-026-01935-x

**Published:** 2026-06-15

**Authors:** Alberto Peraza-Labrador, Justin Bishop, Laura S. Schmidt, John Wright, W. Marston Linehan, George Snipes, Erin Chapman, Doreen Palsgrove, Mathew Kesterke, Michael Keeney, Todd M. Stevens, Madhu Shrestha

**Affiliations:** 1 Department of Diagnostic Sciences, Texas A&M University College of Dentistry, Dallas, TX 75246 USA; 2 Department of Pathology, University of Texas Southwestern Medical Center, Dallas, TX 75390 USA; 3 Urologic Oncology Branch, Center for Cancer Research, National Cancer Institute, National Institutes of Health, Bethesda, MD 20892 USA; 4 Basic Science Program, Frederick National Laboratory for Cancer Research, Frederick, MD 21702 USA; 5 Department of Neuropathology and Surgical Pathology, Baylor University Medical Center, Dallas, TX 75246 USA; 6 Anatomical Pathology, University of Alberta Hospital, Walter Mackenzie Centre, Edmonton, AB T6G 2G3 Canada; 7 Department of Orthodontics, Texas A&M University College of Dentistry, Dallas, TX 75246 USA; 8 Laboratory Medicine and Pathology Anatomic Pathology, Mayo Clinic, Rochester, MN USA; 9 Director of the Head and Neck Pathology Service, The University of Kansas Health System, Kansas, KS USA

**Keywords:** Birt-Hogg-Dubé Syndrome (BHDS), Folliculin (FLCN), Glycoprotein non-metastatic melanoma protein B (GPNMB), Immunohistochemistry, Parotid oncocytomas

## Abstract

**Background:**

Birt–Hogg–Dubé syndrome (BHDS) is a rare autosomal dominant disorder caused by germline pathogenic variations in the *FLCN* gene. It is characterized by cutaneous fibrofolliculomas, pulmonary cysts with risk of pneumothorax, and an increased risk of renal neoplasms. Salivary gland involvement, particularly parotid oncocytomas, is uncommon and may represent an underrecognized manifestation. This study evaluates the immunohistochemical (IHC) expression of folliculin (FLCN) and glycoprotein non-metastatic melanoma protein B (GPNMB) in parotid oncocytomas to assess their potential utility in identifying BHDS-associated lesions.

**Methods:**

A multicenter retrospective study was performed on formalin-fixed, paraffin-embedded specimens, including BHDS-associated parotid oncocytomas, non-syndromic parotid oncocytomas, and BHDS-associated hybrid oncocytic/chromophobe renal tumors (HOCTs) as controls. IHC staining for FLCN and GPNMB was analyzed based on intensity, localization, and distribution. Statistical analysis was performed using chi-square testing.

**Results:**

BHDS-associated parotid oncocytomas occurred in younger patients (n = 4, mean age: 53.7 years) compared to non-syndromic oncocytomas (n = 10; mean age: 72.5 years). Histologically, all syndromic cases demonstrated prominent cytoplasmic clearing and vacuolization on hematoxylin–eosin (H&E) staining, a feature not observed in non-syndromic counterparts (*p* ≤ 0.05). FLCN expression was variable in BHDS-associated parotid oncocytomas, showing patchy nuclear and cytoplasmic staining without statistically significant differences between syndromic and non-syndromic groups (*p* ≥ 0.077). In contrast, GPNMB exhibited strong, diffuse granular cytoplasmic expression in both BHDS-associated renal tumor controls, and parotid oncocytomas, while non-syndromic parotid oncocytomas lacked this pattern (*p* ≤ 0.05).

**Conclusions:**

Parotid oncocytomas in BHDS demonstrate distinctive clinicopathologic features, including younger patient age, cytoplasmic clearing, and a characteristic GPNMB-positive immunophenotype. These findings distinguish them from sporadic parotid oncocytomas and support the use of GPNMB as a potential adjunct marker to raise suspicion for BHDS. Recognition of this association is clinically significant, as patients with BHDS have an estimated 15–30% risk of developing renal malignancies, including chromophobe renal cell carcinoma and clear cell renal cell carcinoma, underscoring the importance of early diagnosis and appropriate surveillance.

## Introduction

Birt-Hogg-Dubé syndrome (BHDS) is an autosomal dominant disorder caused by germline pathogenic variants in the FLCN gene on chromosome 17p11.2 [1, 2]. Although initially described as a genodermatosis characterized by fibrofolliculomas and other benign cutaneous lesions [3–6], the syndrome is also associated with pulmonary cysts, and spontaneous pneumothorax, usually manifesting between the ages of 20 and 30, or even younger [10–12]. BHDS patients have an increased risk for developing renal neoplasms, most often hybrid oncocytic/chromophobe tumors with features of both chromophobe renal carcinoma and oncocytoma (HOCT), and chromophobe renal cell carcinoma [5–13], which generally appear before the age of 50, occurring in up to 30% of the patients [5–9]. In addition, rare salivary gland oncocytomas—predominantly involving the parotid gland, have increasingly been recognized within the syndromic spectrum [14]. Despite their rarity, a systematic review of 147 parotid oncocytomas demonstrated that approximately 8% occurred in BHDS affected individuals, with a mean age of 47 years [15], significantly younger than sporadic oncocytomas.

Of note, Pavlovich et al. reported that renal oncocytomas made up 5% of all kidney neoplasia reported in a BHDS patient cohort [8]. The estimated prevalence of BHDS in the general population was approximately 1.86 per million, with a nearly equal distribution between men (1.86 per million) and women (1.88 per million) [16]. On the other hand, Orphanet, a resource for rare diseases, estimates BHDS prevalence at around 1 in 200,000 individuals [17]. Interestingly, a current study based on evaluation of a health care system population in the U.S. found *FLCN* truncating variants in 1 in 3234 unrelated individuals, 68.7% of whom had at least one clinical feature known to be part of the BHDS-associated phenotype [18] indicating that the syndrome is more common than previously appreciated.

**Fig. 1:**
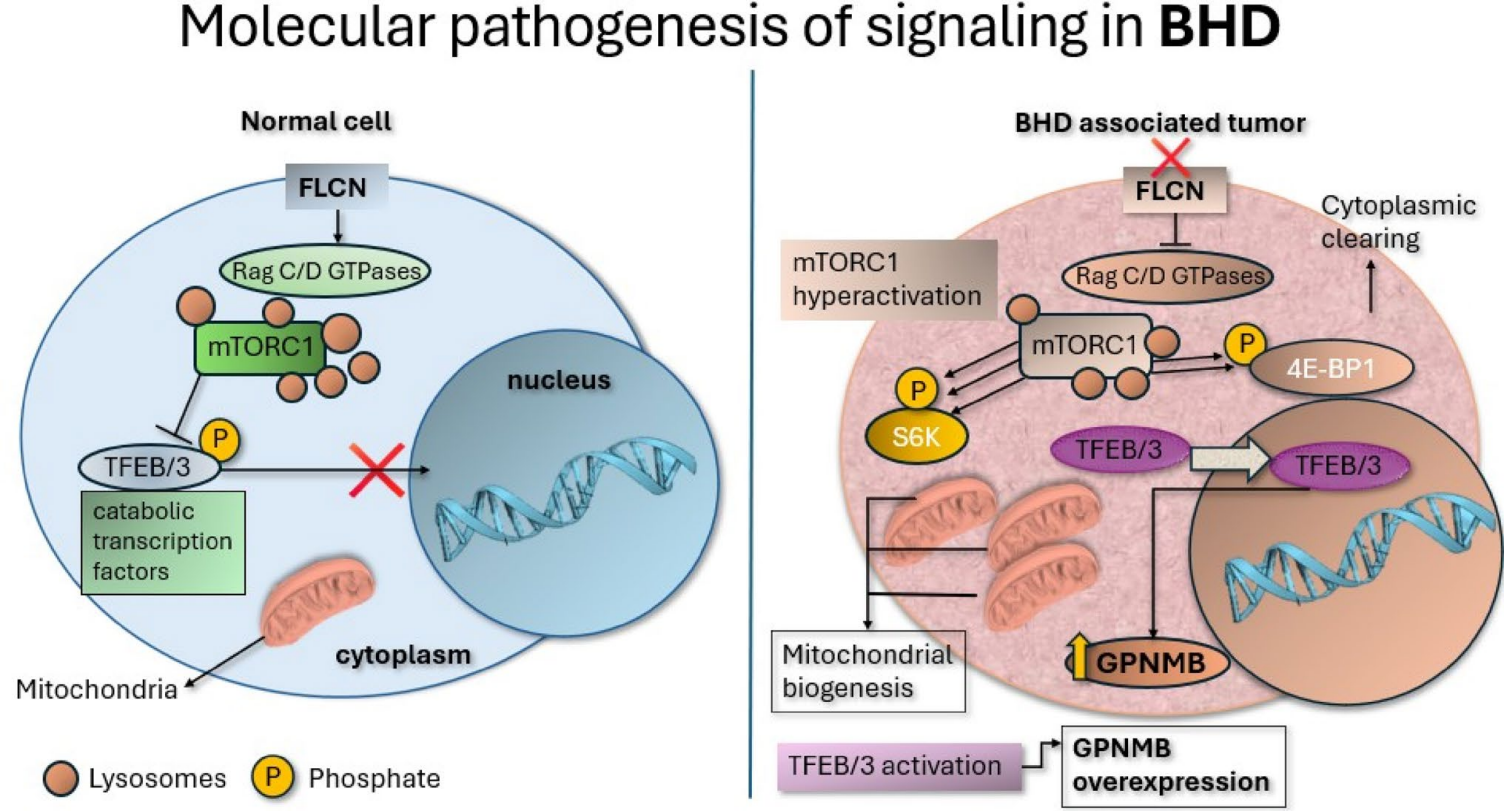
Molecular pathogenesis in BHDS. In normal cells, FLCN acts as a GTPase activating protein (GAP) towards Rag C/D, maintaining them in their GDP-loaded active state. mTORC1 is then recruited to the lysosomal membrane where it can phosphorylate TFEB/TFE3, sequestering these transcription factors in the cytoplasm in their inactive state. Tumors that arise in the setting of BHDS lack functional FLCN due to mutation or loss of both FLCN alleles. Rag C/D remains inactive inhibiting phosphorylation of TFEB/TFE3 by mTORC1. Active TFEB/TFE3 now can translocate to the nucleus where it transcriptionally activates its target genes including GPNMB and RagC/D, resulting in hyperactivation of mTORC1 through its canonical substrates pS6K and p4EBP1

The *FLCN* gene encodes the FLCN protein that functions as a tumor suppressor. FLCN, which binds FLCN-interacting proteins 1 and 2 (FNIP1 and FNIP1) [19, 20], is a bioenergetic (ATP) [21] and nutrient sensor [22]. It is involved in a noncanonical mTOR signaling pathway, that controls cell metabolism, development, and autophagy, as well as PGC1α-driven mitochondrial metabolism through regulation of TFE3 and TFEB function [23]. In addition, the FLCN-FNIP complex plays a role in responding to cellular nutrient status [22] and works in conjunction with AMPK (AMP-activated protein kinase) to regulate energy homeostasis [21, 24] (Fig. 1).

The Glycoprotein non-metastatic melanoma protein B (GPNMB) is a type I transmembrane glycoprotein involved in cellular differentiation and development, localized in the plasma membrane and intracellular compartments, including the cytoplasm [25]. GPNMB plays a role in osteoblast and melanocyte differentiation, is involved in tissue repair and wound healing, and regulates immune responses by modulating macrophage activity. It can have anti-inflammatory effects, particularly in chronic inflammation [26]. In addition, GPNMB is overexpressed in various tumors (e.g., melanoma, glioblastoma, breast cancer), underscoring a role in promoting tumor growth, invasion, and metastasis in certain cancers [27]. GPNMB is a transcriptional target of the transcription factors TFE3 and TFEB, which are activated by loss of FLCN [28–30].

The transcription factor binding to IGHM enhancer 3 (*TFE3*) gene encodes a transcription factor that serves as a key regulator of lysosome biogenesis [31]. The FLCN–FNIP1/2 complex regulates TFE3 and another MiTF family member TFE/B through RagC/D-mediated noncanonical mTORC1 signaling, promoting TFE3 and TFEB cytoplasmic sequestration. Loss of FLCN function results in nuclear localization and transcriptional upregulation of TFE3/B downstream targets, including GPNMB, potentially contributing to altered lysosomal and metabolic signaling [23, 31–33].

This study aims to evaluate the immunohistochemical expression of FLCN, and GPNMB, a readout for TFE3 transcriptional activation, in parotid oncocytomas as a potential screening tool to aid in the diagnosis of Birt-Hogg-Dubé syndrome (BHDS).

## Methodology

### Ethics

This study was conducted following ethical standards and guidelines, with approval from the Texas A&M University College of Dentistry Research Ethics Committee (IRB2024-1488), following the Declaration of Helsinki. To maintain confidentiality and reduce potential bias, all case data were anonymized and labeled with unique identifiers before histological and immunohistochemical evaluation.

### Test Cases and Controls

This retrospective multi-center study compared the immunohistochemical staining patterns of FLCN and GPNMB in BHDS-associated and non-syndromic parotid oncocytomas, using hybrid oncocytic chromophobe renal tumors (HOCT) as a control cohort. Notably, the present series includes four previously unpublished BHDS-associated parotid oncocytomas, significantly expanding the number of molecularly characterized cases currently documented in the literature.

Five HOCT tumors from BHDS patients managed by the Urologic Oncology Branch, National Cancer Institute, National Institutes of Health, Bethesda, Maryland, served as positive controls. Inclusion criteria required molecular confirmation of the samples associated with BHDS, without restrictions regarding time, race, sex, or age. Ten non-syndromic parotid oncocytomas, nine from Baylor Medical University Center in Dallas, Texas, and one from Mayo Clinic Rochester, served as test cases. *FLCN* mutation analysis was not performed on these cases; however, according to the case histories, the patients had a mean age at diagnosis of 72.5 years with no history of skin, lung or kidney manifestations. Three BHDS-associated parotid oncocytomas from the University of Alberta (Canada), and one case from Kansas University, Department of Pathology, were used as test cases.

### Hematoxylin and Eosin Evaluation of Parotid Oncocytomas

All cases were independently reviewed and analyzed by one head and neck pathologist (JB) and two board-certified oral and maxillofacial pathologists (OMFPs JW and MS). Cases were excluded if there was disagreement with the initial diagnosis. Each variable was added as dichotomous information (YES/NO), checking for clear cells, and the intensity of clearing. A semi quantitative score system was employed using Microsoft Excel 2022 (Microsoft Corp., Redmond, WA, USA) that measures the percentage of tumor cells and the proportion of the stain intensity analyzed (Table 1). Statistical analysis was performed with SPSS (version 23) software (IBM, Chicago, IL, USA) using a chi-square test between categorical. Statistical significance was set at *p* < 0.05.

**Table 1:** Comparative clinicopathologic and immunohistochemical features of BHDS-associated parotid oncocytomas, non-syndromic parotid oncocytomas, and hybrid oncocytic chromophobe renal tumors (HOCT)

Variable	BHDS parotid oncocytoma	Non-syndromic oncocytoma	BHDS renal HOCT	*p*-value
Mean age (Years)	53.7	72.5	49.6	*p* ≤ 0.05
Clear cells (%)	80	10	90	*p* ≤ 0.05
Perinuclear halos (%)	75	5	80	*p* ≤ 0.05
Mean FLCN score (%)	25	100	15	*p* ≤ 0.077
Mean GPNMB score (%)	100	15	90	*p* ≤ 0.05

BHDS-associated tumors demonstrated a higher frequency of clear cell change and perinuclear halos, as well as significantly increased GPNMB expression compared with non-syndromic oncocytomas. FLCN expression showed lower mean scores in BHDS-associated tumors without reaching statistical significance

### Selection of Antibodies

The antibodies used for immunohistochemistry were polyclonal, which are composed of a variety of B cell clones that bind to multiple or different epitopes [34]. They include FLCN (Rabbit polyclonal FLCN antibody ABCAM ab176707) and Human Osteoactivin/GPNMB. (Goat Polyclonal Biotechne R&D systems AF2330). The antibody selection was based on published studies evaluating the expression of FLCN [35] and GPNMB [44] in FLCN-deficient cases.

### Immunohistochemical Staining

Sections from formalin-fixed and paraffin-embedded tissue were cut at 4 µm thickness and mounted on unstained glass slides for IHC. The sections were deparaffinized by placing them in an incubator at 56 °C for an hour, followed by two changes of xylene for 10 min twice. Rehydration was performed by two changes of 100% ethanol for 5 min and 3 min, respectively, followed by 95% ethanol for 2 min, 70% and 50% ethanol for 2 min each, and finally, distilled water (ddH2O) for 2 min. The antigen retrieval (Leica, pH 6 Citrate buffer) was incubated in a steamer at 95/100 °C for 30 min. After washing with PBST, the slides were incubated in 3% hydrogen peroxide for 10 min in the dark. The sections were blocked with 3% BSA and 10% normal goat serum in 1xPBST, 100 µl/section for 2 h at room temperature. The primary antibody was added at 100 µl per slide, diluted in 2% goat serum, and incubated at 4 °C overnight (24 h). The secondary antibody, consisting of 1:200 biotinylated goat anti-mouse IgG (Vector BA-2000) in 2% horse serum/PBST or 1:200 biotinylated goat anti-rabbit IgG (Vector BA-1000) in 2% goat serum/PBST, was added at room temperature for 1 h. The ABC reagent VECTASSTAIN ABC-HRP kit (Vector PK-4002) was used, mixing A&B 1:100 in 2% horse serum/PBST or 2% goat serum/PBST at room temperature for 1 h. For color development, the DAB kit, Vector SK-4105, was added in 30-µl increments and incubated in DAB solution until the color developed. The sample was then rinsed in distilled water and counterstained with Methyl green. The slides were dehydrated with 80% EtOH for 10 s, then 100% EtOH for 2 min twice, cleared with xylene, and mounted. Brownish cytoplasmic and nuclear staining was considered positive, regardless of the intensity. Brownish cytoplasmic staining was considered positive, regardless of the intensity.

### Scoring

The proportion of oncocytic cells exhibiting nuclear and/or cytoplasmic expression of FLCN and GPNMB proteins was assessed based on staining intensity and distribution. Expression was categorized as weak (1–5%), moderate (5–10%), or strong (> 10%) localized in the nucleus, cytoplasm, or both. A percentage of positive cells was determined to evaluate the pattern of IHC. Quantification was based on the average number of positively stained cells across 10 high-power fields per specimen, evaluated in non-inflamed areas under light microscopy with evaluation of the intensity of the expression in the tissue as 0–100%.

### Statistical Analysis

Descriptive statistics for categorical variables were reported as frequencies and percentages. Given the small sample size and categorical nature of the data, chi-square tests were used to assess statistical significance (*p* ≤ 0.05) between observed and expected outcomes for FLCN and GPNMB. All analyses were performed using SPSS version 29.0 (Armonk, NY).

## Results

**Fig. 2:**
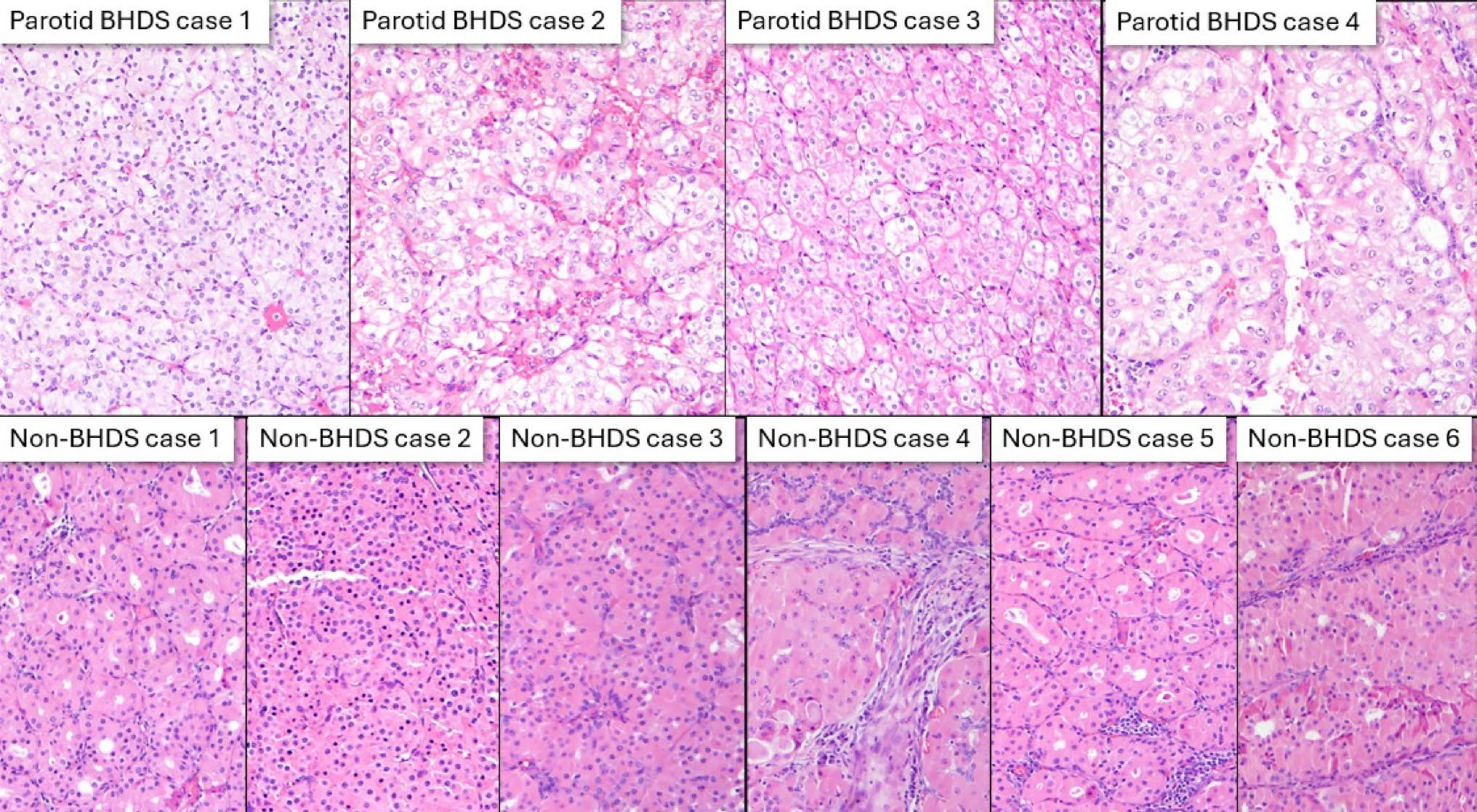
H&E staining of clear cell oncocytomas of the salivary gland in four BHDS patients (10x). Clearly demarcated solid tumors are shown. The syndromic parotid tumors (upper panels) are composed of oncocytic cells with peri-nuclear halos, compared to H&E-stained Sects. (20x) from 6 of 10 non-syndromic parotid oncocytoma cases (lower panels) demonstrating classic oncocytic morphology, characterized by nests and sheets of cells with abundant granular eosinophilic cytoplasm and uniform round nuclei, without significant cytoplasmic clearing

**Fig. 3:**
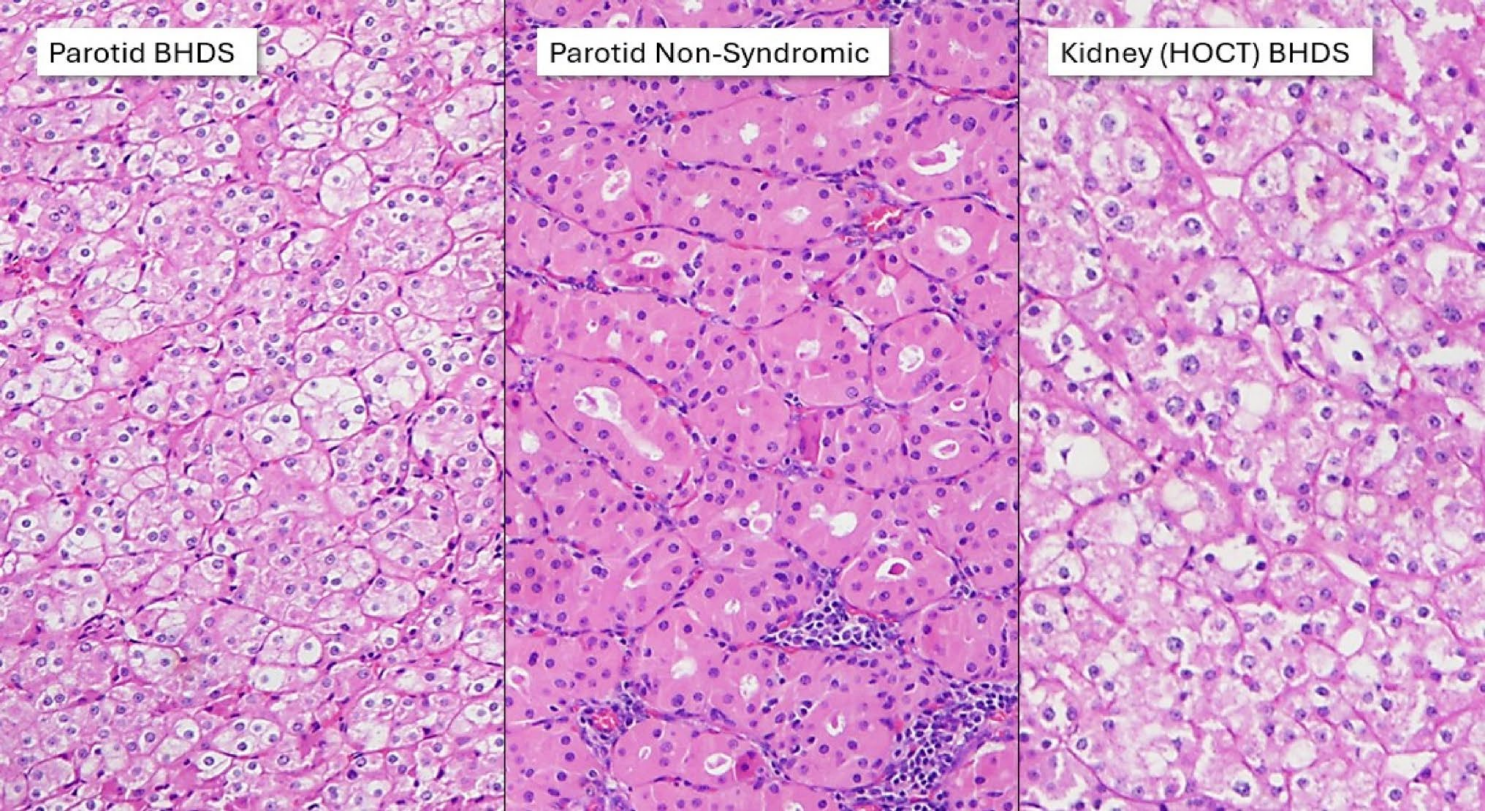
Representative H&E-stained Sects. (20x) of BHDS-associated parotid oncocytoma (left) and renal hybrid oncocytic/chromophobe tumor (HOCT) (right) demonstrating prominent cytoplasmic clearing and vacuolization. In contrast, sporadic (non-syndromic) parotid oncocytoma (center) shows conventional oncocytic morphology composed of large, polygonal epithelial cells (oncocytes) with abundant granular, eosinophilic cytoplasm (*p* ≤ 0.05)

H&E staining was carried out to compare histologic/morphologic differences between the test and control cases. All 4 BHDS-associated parotid oncocytomas (Fig. 2) and the BHDS control cohort (HOCT) (Fig. 3) exhibited consistent morphologic features characterized by prominent clear cells and cytoplasmic vacuolations, demonstrating a statistically significant difference from non-syndromic parotid oncocytomas (Figs. 2 and 3) by chi-square test (*p* ≤ 0.05).

**Fig. 4:**
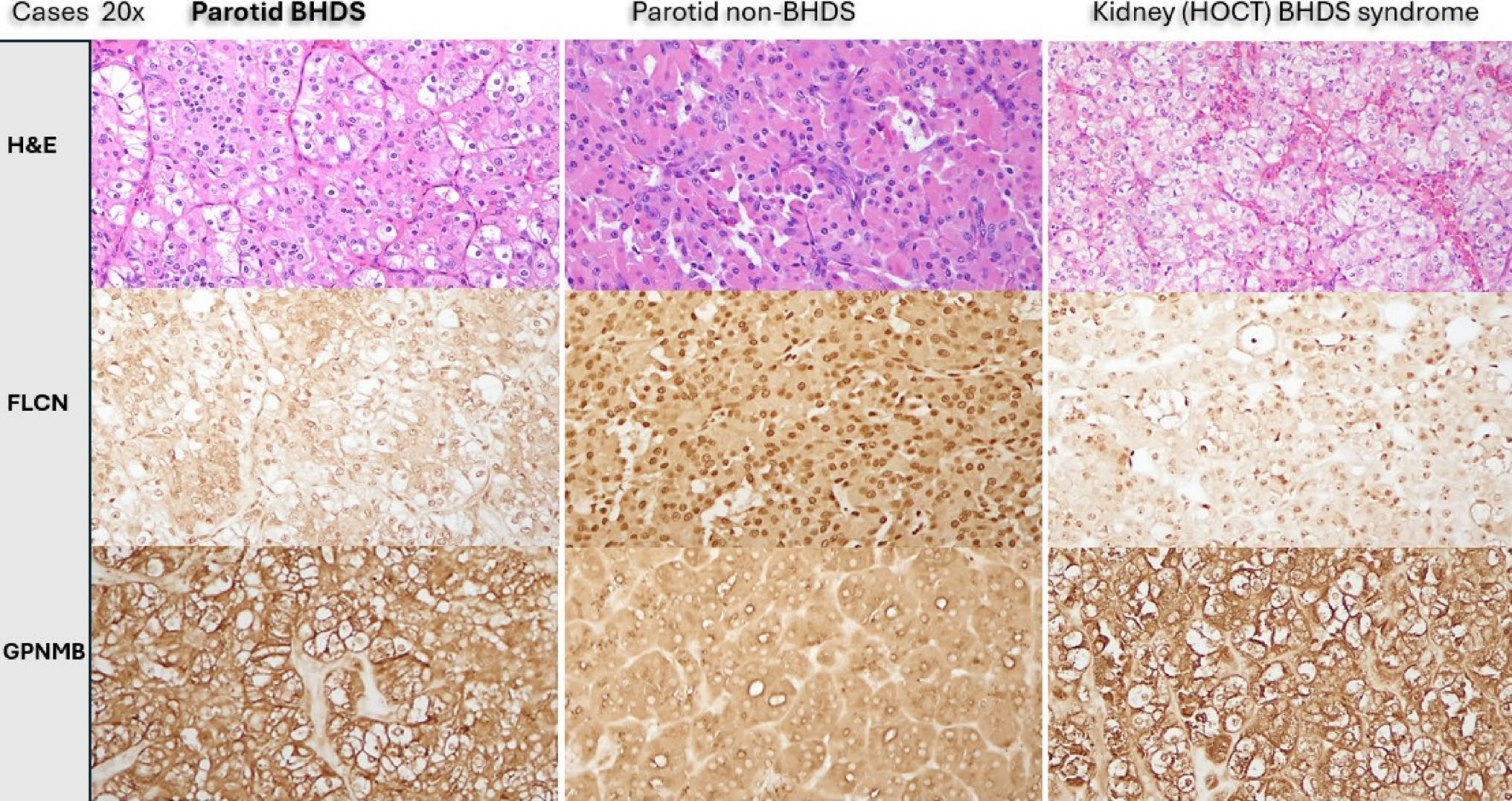
H&E-stained Sects. (20x) of BHDS-associated parotid oncocytoma and control cohort compared with sporadic parotid oncocytoma, demonstrating prominent cytoplasmic clearing and vacuolization in BHDS-associated tumors, a statistically significant finding (*p* ≤ 0.05). FLCN IHC showing variable nuclear and cytoplasmic expression without statistically significant differences between groups (*p* ≤ 0.077); sporadic oncocytoma demonstrates strong nuclear and cytoplasmic staining. GPNMB IHC showing strong granular cytoplasmic expression in BHDS-associated tumors, with absent or minimal expression in sporadic oncocytoma, representing a statistically significant difference (*p* ≤ 0.05)

**Fig. 5:**
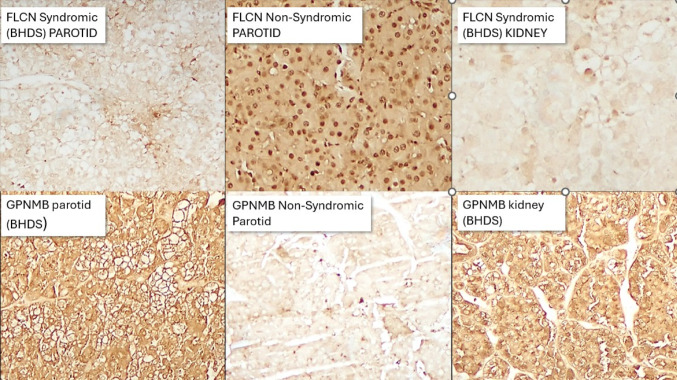
Representative FLCN IHC with semi-quantitative scoring based on the proportion of positive tumor cells and staining intensity, as indicated. BHDS-associated parotid oncocytomas (upper left) and HOCT tumors (upper right) demonstrate variable, patchy nuclear and cytoplasmic FLCN expression with lower composite scores, whereas sporadic parotid oncocytomas show more diffuse and stronger nuclear and cytoplasmic FLCN staining, resulting in higher scores (upper center). Representative GPNMB IHC with semi-quantitative scoring based on the proportion of positive tumor cells and staining intensity, as indicated. BHDS-associated parotid oncocytomas (lower left) and HOCT tumors (lower right) demonstrate strong, diffuse granular cytoplasmic expression with higher composite scores, whereas sporadic parotid oncocytomas show absent or minimal staining with lower scores (lower center). The difference in GPNMB expression between groups was statistically significant (*p* ≤ 0.05)

IHC staining of FLCN and GPNMB, well established negative and positive markers for BHDS respectively, was performed to further aid in distinguishing syndromic from non-syndromic parotid oncocytoma cases. IHC analysis of FLCN showed variable expression in BHDS-associated parotid oncocytomas and control cohort, characterized by patchy nuclear and cytoplasmic staining involving approximately 15–30% of tumor cells, with mild to moderate intensity (Table 1 and Fig. 4). This pattern did not reach statistical significance when compared to non-syndromic cases (*p* ≥ 0.07). In contrast, 75% of non-syndromic oncocytomas demonstrated diffuse strong nuclear and cytoplasmic FLCN expression in nearly 100% of tumor cells (Table 1 and Fig. 5).

Expression of GPNMB, however, showed a strong association with BHDS status (*p* < 0.05) (Figs. 4 and 5). Both BHDS parotid oncocytomas and BHDS-associated renal tumors exhibited diffuse and strong staining, involving ≥ 85% of tumor cells. In contrast, non-syndromic oncocytomas demonstrated limited GPNMB expression, with mild intensity staining observed in only 15–20% of tumor cells (Table 1 and Fig. 5).

**Fig. 6:**
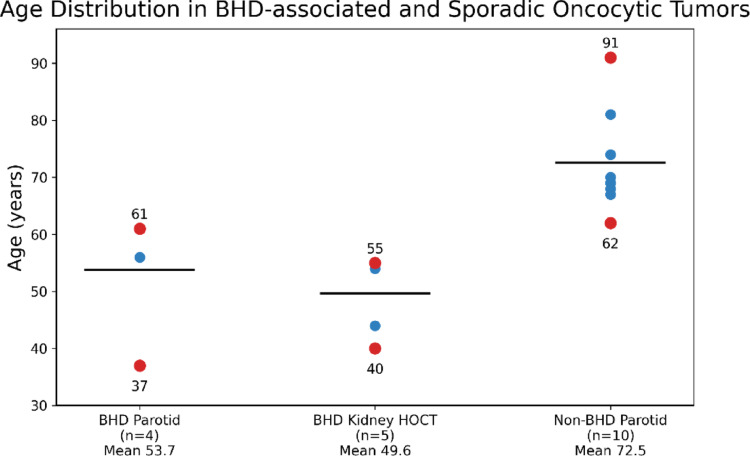
Age distribution of BHDS-associated and sporadic parotid oncocytic tumors. Scatter plot showing individual patient ages for BHDS-associated parotid oncocytomas, associated with control cohort and non-BHDS parotid oncocytomas. Black horizontal lines indicate mean age for each group. BHD-associated tumors present at a younger age compared to sporadic parotid oncocytomas

Another distinguishing feature was the difference in age of onset of parotid oncocytomas in the two groups. The mean age of diagnosis for patients with BHDS-associated parotid tumors was 53.7 years, while BHDS patients were diagnosed with HOCT tumors at a mean age of 49.6 years. In contrast, patients with non-syndromic oncocytomas were diagnosed at a significantly older age, with a mean age of 72.5 years (Fig. 6).

## Discussion

BHDS-associated parotid oncocytomas exhibited a distinctive immunohistochemical profile compared to non-syndromic oncocytomas. Patchy and diffuse FLCN expression in both nucleus and cytoplasm of BHDS oncocytomas may support the impact of *FLCN* mutations on protein mislocalization and tumorigenesis. GPNMB strong positivity is an indicator of TFE3/TFEB transcriptional activation and may reflect altered mitochondrial or lysosomal biology, consistent with oncocytic differentiation, serving as a useful adjunct marker for BHDS.

In this study, we have shown that parotid oncocytomas from patients with BHDS demonstrate a distinctive clear-cell phenotype that is statistically significant (*p* < 0.05) when compared with non-syndromic parotid oncocytomas. The presence of multifocal or patchy cytoplasmic clearing, often more pronounced in specific regions of the tumor, was similar in morphologic appearance to the control cohort (HOCT), used as positive controls. This morphological overlap reinforces the concept that BHDS-associated oncocytic neoplasms across different organ systems share a common histopathologic signature driven by FLCN pathway dysregulation. In this context, this pattern underscores a distinct molecular signature in BHDS oncocytomas, differentiating them from sporadic parotid oncocytomas and highlighting potential diagnostic and biological implications.

Importantly, the degree and distribution of cytoplasmic clearing in BHDS-associated parotid oncocytomas were qualitatively and quantitatively distinct from the clearing occasionally seen in sporadic oncocytomas. In non-syndromic cases, clearing is usually either minimal, focal, or attributable to artifacts such as degenerative change or fixation differences [15, 39]. In this study, the cytoplasmic clearing observed in BHDS-associated parotid oncocytomas may reflect a metabolic and structural phenotype analogous to BHDS-associated renal oncocytic tumors, in which FLCN loss has been linked to mitochondrial and metabolic dysregulation [40]. These findings support the idea that clear-cell changes in oncocytic salivary tumors should raise clinical suspicion for BHDS, particularly in patients with personal or family histories of early-onset renal tumors, especially chromophobe or HOCT subtypes, early onset spontaneous pneumothorax, or cutaneous fibrofolliculomas [6]. Interestingly, one of the parotid tumors was initially evaluated as a potential metastasis from a renal neoplasm; however, review of the patient's medical history revealed a diagnosis of BHDS. This parotid oncocytoma demonstrated focal, patchy PAX8 expression, a finding of potential diagnostic significance because PAX8 positivity in a clear cell salivary gland neoplasm may raise concern for metastatic renal cell carcinoma. Similar PAX8 expression was recently reported by Alexander et al. in a BHDS-associated clear cell parotid oncocytoma occurring in a 30-year-old patient without evidence of renal metastasis [46]. In contrast, the remaining BHDS-associated parotid oncocytomas in the present study were negative for PAX8.

The clear cell morphology observed in these tumors raises the possibility of glycogen accumulation as a potential mechanism; In histologic methods to detect glycogen in clear cell renal cell carcinoma, the tumor tissue is digested with the diastase enzyme, which breaks down glycogen into soluble maltose and glucose that are washed away [42]. Although PAS-positive, diastase-sensitive cytoplasmic material consistent with glycogen was identified in most BHDS-associated tumors evaluated. The clear cell phenotype observed in these neoplasms may reflect broader metabolic dysregulation related to FLCN deficiency. FLCN dysfunction has been linked to altered mTOR signaling, mitochondrial homeostasis, and cellular energy sensing pathways, which may contribute to the distinctive hybrid oncocytic-clear cell morphology characteristic of BHDS-associated neoplasms [41]. In this context, Isono et al. demonstrated that FLCN deficiency, targeted to the salivary gland in a genetically engineered mouse model, drives metabolic reprogramming (glycolysis and pentose phosphate pathway), promoting nucleotide synthesis and cellular proliferation in *FLCN* deficient salivary glands [43].

Although FLCN IHC was included in our panel, the staining did not show a statistically significant correlation with BHDS-associated parotid oncocytoma cases. This aligns with prior reports noting that loss of FLCN expression may be inconsistent or difficult to interpret in formalin-fixed specimens, likely due to technical variability and the complex biology of FLCN protein expression [35]. Previous studies have reported variable and sometimes non-reproducible FLCN immunohistochemical expression in BHDS-associated renal tumors, particularly when different antibodies and formalin-fixed, paraffin-embedded tissues were evaluated [35]. Similarly, Zhao et al. reported only limited FLCN expression in clear cell renal cell carcinoma [36], although nuclear accumulation of FLCN has been described in normal cells [37, 38]. In contrast, GPNMB has emerged as a more consistent marker in BHDS-associated renal neoplasms, with studies demonstrating GPNMB overexpression accompanied by reduced or variable FLCN expression [29, 30]. Therefore, based on our data, FLCN IHC is not a reliable immunomarker for use as a diagnostic tool for salivary gland pathology. In contrast, GPNMB expression demonstrated a statistically significant association (*p* ≤ 0.05) with BHDS-associated parotid oncocytomas when compared with non-syndromic oncocytomas. This finding is noteworthy because GPNMB has been reported as a potential surrogate marker of FLCN pathway dysregulation in FLCN-mutated renal HOCT tumors and other FLCN-mutated oncocytic neoplasms [44]. However, despite its potential utility, we emphasize that GPNMB staining must be interpreted cautiously. Expression is not entirely specific, can be seen in other oncocytic lesions, and should be evaluated in the context of morphology, clinical history, and—when possible—genetic confirmation of a germline *FLCN* mutation.

Importantly, mosaicism may also contribute to the pathogenesis of BHDS and the development of FLCN-mutated tumors in tissues other than kidney including the parotid glands. Although most cases arise from a germline *FLCN* mutation, rarely, early de novo mutations may also result in mosaic distribution, underscoring the genetic complexity of BHD-associated tumorigenesis. However, to date evidence of mosaicism in BHDS has not been reported.

In a research or clinical diagnostic laboratory setting, definitive test could be performed to confirm that FLCN loss was causative for parotid oncocytoma development. DNA extracted from formalin-fixed paraffin-embedded parotid tissues could be sequenced to look for a *FLCN* second hit alteration and show that both copies of FLCN in the parotid oncocytoma were lost or mutated. This approach could be pursued in follow-up studies of these BHDS parotid oncocytoma cases. Taken together our findings highlight a reproducible morphologic pattern—prominent clear-cell change—and a supportive immunohistochemical marker—GPNMB positivity—that can assist in recognizing BHDS-associated parotid oncocytomas. While no single feature is diagnostic, the combination of (1) onset of parotid oncocytomas in patients younger than 55 years of age, (2) oncocytic morphology with clear-cell areas, and (3) GPNMB IHC expression should prompt pathologists to consider the possibility of BHDS and recommend appropriate clinical or genetic evaluation. In this context, abdominal imaging is recommended at least every three years as part of clinical surveillance for patients with BHDS [45]. In this regard, a major strength of the present study is the inclusion of four previously unpublished BHDS-associated oncocytoma cases, thereby substantially increasing the number of molecularly and histologically characterized cases currently available in the literature.

## Conclusion

Our study has shown for the first time that BHDS parotid oncocytomas show unique morphology with significant cytoplasmic clearing and GPNMB positivity compared to non-syndromic parotid tumors. This finding should alert pathologists/clinicians to further investigate clear cell or partially clear cell parotid oncocytomas in patients younger than 55 with strong cytoplasmic GPNMB expression. In such cases, genetic counseling should be advised.

## Data Availability

No datasets were generated or analysed during the current study.

## Code Availability

Statistical Package for the Social Sciences (SPSS) software, version 20.0, ©Copyright IBM (SPSS Inc., Chicago, IL, USA).
